# InSb-added TiO_2_ nanocomposite films by RF sputtering

**DOI:** 10.1186/1556-276X-8-269

**Published:** 2013-06-07

**Authors:** Seishi Abe

**Affiliations:** 1 Research Institute for Electromagnetic Materials, Sendai 982-0807, Japan

**Keywords:** TiO_2_ nanocomposite, Band gap, RF sputtering

## Abstract

This study investigates the preparation of InSb-added TiO_2_ nanocomposite films by RF sputtering. The optical absorption spectra are obviously shifted to visible and near-infrared regions. High-resolution transmission electron microscopy indicates that sphere-shaped InSb nanocrystals with a size of about 15 nm are dispersed in a matrix. The X-ray diffraction result reveals that the matrix forms a phase mixture of TiO_2_ and In_2_O_3_, which is also produced by decomposing the InSb during postannealing at 723 K. Therefore, the absorption shift is clearly due to quantum size effects of the InSb nanocrystals embedded in the wide-gap oxides TiO_2_ and In_2_O_3_.

## Background

Quantum dot solar cells have attracted much attention because of their potential to increase conversion efficiency [1]. Specifically, the optical absorption edge of a semiconductor nanocrystal is often shifted due to quantum size effects. The optical band gap can then be tuned to an effective energy region for absorbing the maximum intensity of the solar radiation spectrum. Furthermore, quantum dots produce multiple electron–hole pairs per photon through impact ionization, whereas bulk semiconductor produces one electron–hole pair per photon.

A wide-gap semiconductor sensitized by semiconductor nanocrystals is a candidate material for such use. Wide-gap materials such as TiO_2_ and ZnO can only absorb the ultraviolet (UV) part of the solar radiation spectrum. The semiconductor nanocrystal supports the absorption of visible (vis) and near-infrared (NIR) light. Up to now, various nanocrystalline materials (InP [2], CdSe [3], CdS [4,5], PbS [6], and Ge [7,8]) have been investigated as sensitizers for TiO_2_. Wide-gap semiconductor ZnO was also investigated, since the band gap and the energetic position of the valence band maximum and conduction band minimum of ZnO are very close to those of TiO_2_[9]. Most of these composite materials were synthesized through chemical techniques, although physical deposition, such as sputtering, is also useful. In addition, one-step synthesis of a composite thin film is favorable for low-cost production of solar cells. Package synthesis requires a specific material design for each deposition technique, for example, radio frequency (RF) sputtering [10,11] and hot-wall deposition [12]. The present study proposes a new composite thin film with InSb-added TiO_2_ produced by RF sputtering. InSb nanocrystals may exhibit relatively high absorption efficiency due to a direct band structure with 0.17eV [13] and an exciton Bohr radius of 65.5 nm [14]. According to the material design, based on differences in the heat of formation [10,11], InSb nanocrystals are thermodynamically stable in an TiO_2_, since Ti is oxidized more than InSb because the free energy of oxidation in InSbO_4_, which is a typical oxide of InSb, exceeds that of the TiO_2_[15,16]. In addition, nanocrystalline InSb dispersed in the oxide matrix may exhibit quantum size effects, due to the wide band-gap of 3.2 eV in TiO_2_ with anatase structure [17]. However, it is difficult to forecast how the composite will be formed in the one-step synthesis, since the compound semiconductor, InSb, may have decomposed during the preparation process. In the current study, the composition of InSb-added TiO_2_ nanocomposite film is varied widely to find a composite with vis-NIR absorption due to the presence of InSb nanocrystals embedded in the wide-gap oxide matrix.

## Methods

An InSb-added TiO_2_ nanocomposite film was prepared by RF sputtering from a composite target. Specifically, 5 × 5 mm^2^ InSb chips, which were cleaved from a 2-in diameter InSb (100) wafer, were set on a 4-in diameter ceramic TiO_2_ target. The chamber was first evacuated to a vacuum of 1.5 × 10^−7^ Torr. InSb-added TiO_2_ nanocomposite films were deposited on a Corning #7059 glass substrate (Norcross, GA, USA) cooled by water. The distance between the target and the substrate was kept constant at 73 mm. The total gas pressure of argon or argon with diluted oxygen was fixed at 2.0 × 10^−3^ Torr. RF power and deposition time were kept constant at 200 W and 60 min, and no RF bias was applied to the substrate. The InSb-added TiO_2_ nanocomposite films thus deposited were successively annealed at temperatures from 623 to 923 K in 50 K steps for 60 min in a vacuum to crystallize both InSb and TiO_2_. The film was structurally characterized using X-ray diffraction (XRD, Rigaku RAD-X, Rigaku Corporation, Tokyo, Japan). The optical-absorption spectrum of the film was measured using UV–vis-NIR spectroscopy (Shimadzu UV3150, Nakagyo-ku, Kyoto, Japan), and the composition of the film was analyzed using energy-dispersion spectroscopy (EDAX Phoenix, NJ, USA), operating at 10 kV with standard samples of MnTiO_3_ to calibrate the analyzed results for elements Ti and O and with InSb for elements In and Sb. The nanoscale structure was observed using high-resolution transmission electron microscopy (HRTEM, Hitachi H-9000NAR, Hitachi, Ltd., Tokyo, Japan) operating at 300 kV. Ion milling was performed during sample preparation.

## Results and discussion

**Figure 1:**
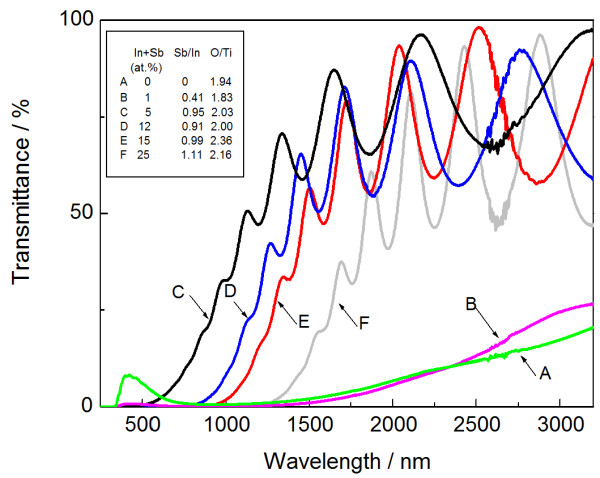
**Optical transmittance spectra of as-deposited InSb-added TiO**_**2 **_**thin films.** Inset indicates EDS analysis results of In + Sb, Sb/In, and O/Ti.

Figure 1 depicts the transmittance spectra of as-deposited InSb-added TiO_2_ thin films prepared in a pure argon atmosphere. The composition of InSb can be varied by employing different InSb chip numbers while keeping almost stoichiometric InSb at concentrations exceeding 5 at.% (In + Sb). At 0 at.% (In + Sb), the optical absorption edge of TiO_2_ is observed at approximately 400 nm, with relatively less optical transparency in a wide range from UV to NIR. This weak transparency is due to the oxygen deficit in TiO_2_ with a composition ratio O/Ti of 1.94. A slight addition of 1 at.% also exhibits similar behavior, but further concentrations exceeding 5 at.% abruptly improve the transparency due to the excess oxygen in TiO_2_ with ratios O/Ti exceeding 2. This result suggests that the oxygen deficit in TiO_2_ is improved by adding InSb. In addition, the optical absorption edge shifts towards the longer wavelength region as the In + Sb content increases.

**Figure 2:**
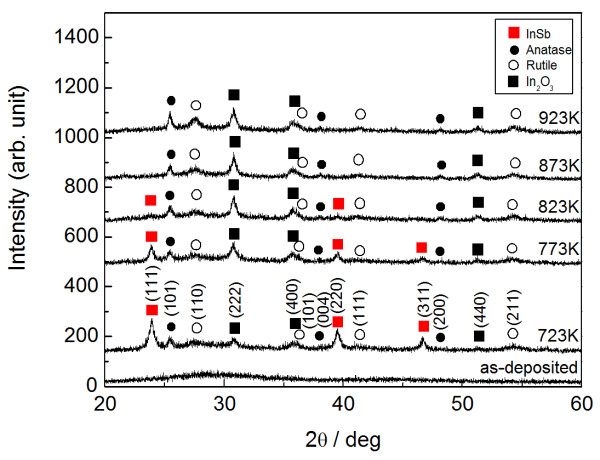
**XRD pattern for InSb-added TiO**_**2 **_**thin films with different annealing temperatures.** Red squares indicate InSb, black squares indicate In_2_O_3_, dots indicate TiO_2_ with anatase structure, and circles indicate TiO_2_ with rutile structure.

Figure 2 presents a typical XRD pattern of InSb-added TiO_2_ thin films annealed at different temperatures. In this case, the film was prepared in pure argon with an InSb chip number of 8 (15 at.% (In + Sb) in as-deposited film). The as-deposited film forms an amorphous structure, with XRD peaks of InSb, In_2_O_3_, and TiO_2_ (anatase and rutile) at a temperature of 723 K. The XRD peak of InSb tends to disappear at temperatures exceeding 823 K, beyond the melting point of 803 K, in InSb [18]. Thus, an annealing temperature of 723 K seems to be better to ensure the InSb phase stability.

**Figure 3:**
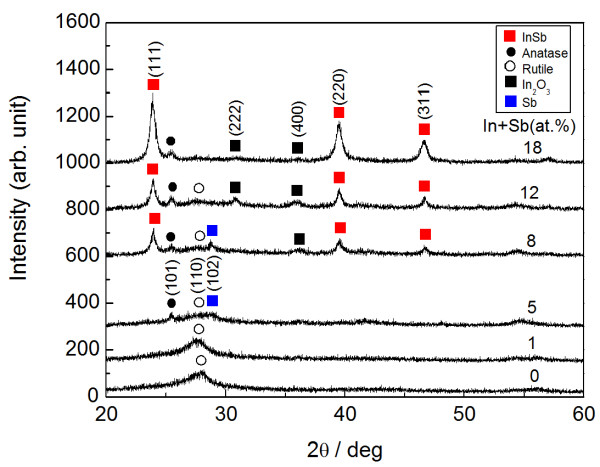
**XRD pattern for InSb-added TiO**_**2 **_**thin films with different In + Sb concentrations.** Red squares indicate InSb, black squares indicate In_2_O_3_, blue squares indicate Sb, dots indicate TiO_2_ with anatase structure, and circles indicate TiO_2_ with rutile structure.

Figure 3 presents the XRD patterns of InSb-added TiO_2_ thin films with different In + Sb concentrations. In this case, the film was deposited in a pure argon atmosphere and subsequently annealed at 723 K. Postannealing reduces the composition of In + Sb in most of the samples, typically from 25 at.% (as-deposited) to 18 at.% (annealed). There are no ternary or quaternary compounds in the patterns. At 0 and 1 at.% (In + Sb), only a rutile structure can be observed, with anatase structure and Sb peaks at 5 at.%, and with InSb and In_2_O_3_ peaks at 8 at.%. Further addition of 12 at.% induces the disappearance of the Sb peak. In the experiment setup, two compounds, InSb and TiO_2_, are employed as the targets (i.e., metal Sb and In_2_O_3_ compound are not used). In addition, the high transparency (Figure 1) strongly suggests that residual metal elements In and Sb are negligible in the as-deposited films with concentrations exceeding 5 at.%. Both Sb and In_2_O_3_ are thus produced by decomposing the added InSb during postannealing.

**Figure 4:**
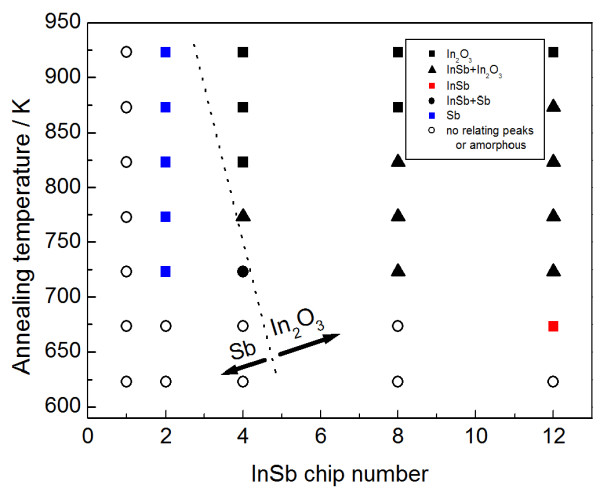
**Relation between InSb-originating phases (InSb, Sb, and In**_**2**_**O**_**3**_**), annealing temperature, and InSb chip number.** Black squares indicate single-phase In_2_O_3_; triangles indicate a phase mixture of InSb and In_2_O_3_; the red square indicates single-phase InSb; dots indicate a phase mixture of InSb and Sb, and circles indicate no relating peaks or amorphous. The dotted line indicates dominant phase change from Sb to In_2_O_3_.

**Figure 5:**
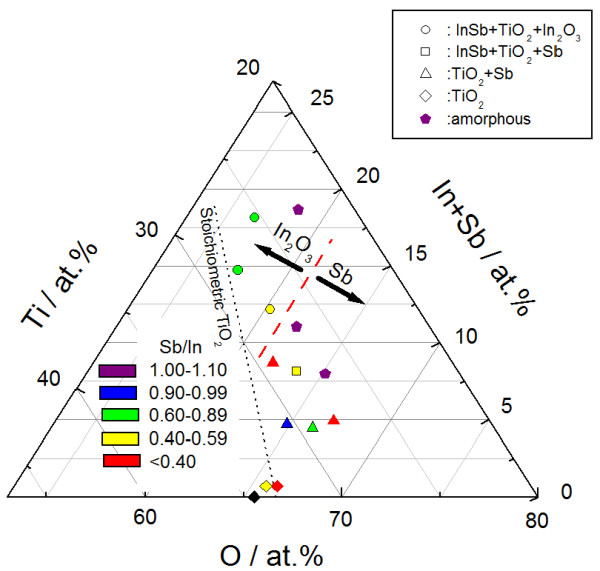
**Compositional plane of phase appearance in InSb-added TiO**_**2 **_**thin films.** Dots indicate a phase mixture of InSb, TiO_2_, and In_2_O_3_; squares indicate a phase mixture of InSb, TiO_2_, and Sb; triangles indicate a phase mixture of TiO_2_ and Sb; rhombuses indicate single-phase TiO_2_; and pentagons indicate amorphous. Violet indicates an Sb/In ratio of 1.00 to 1.10; blue indicates 0.90 to 0.99; green indicates 0.60 to 0.89; yellow indicates 0.40 to 0.59; and red indicates less than 0.40.

The two phases, Sb and In_2_O_3_, are thus produced, due to decomposition of the added InSb during postannealing. These InSb-originating phases (InSb, Sb, and In_2_O_3_) are summarized in Figure 4 with respect to the InSb chip numbers and the annealing temperatures. The InSb phase crystallizes first at 623 K with an InSb chip number of 12 (25 at.% (In + Sb) in the as-deposited film). The Sb phase tends to appear with relatively small InSb chip numbers, less than four chips (12 at.% (In + Sb)), in contrast to the In_2_O_3_ phase with its higher chip numbers and relatively high temperatures. The dominant phase changes from Sb to In_2_O_3_ with respect to the InSb contents and annealing temperatures, although added InSb is almost stoichiometric, 2.7 at.% In + 2.6 at.% Sb with two InSb chips and 7.5 at.% In + 7.5 at.% Sb with eight chips, for example. Next, the composition is varied widely, with Ar and additional oxygen atmosphere, regardless of whether the TiO_2_ phase, which is also contained in the composite, affects the difference in phase appearance (Sb and In_2_O_3_). Figure 5 depicts the compositional plane of the phase appearance in InSb-added TiO_2_ thin films annealed at 723 K. The stoichiometric composition of TiO_2_ with InSb is indicated by a dotted line. Single-phase TiO_2_ appears in relatively low InSb concentrations. In particular, pure TiO_2_ (In + Sb = 0) has an oxygen deficit from stoichiometry in TiO_2_. This deficit causes low optical transparency over a wide wavelength range (Figure 1) at 0 at.% (In + Sb). In contrast, addition of InSb tends to provide excess oxygen from stoichiometric TiO_2_, in accordance with improving the transparency (Figure 1). InSb phase appears at 8 at.% (In + Sb), especially with In_2_O_3_ exceeding 12 at.%. Further addition of oxygen provides an amorphous structure. Although the as-deposited films contain almost stoichiometric InSb, with the Sb/In ratio ranging from 0.9 to 1.2, postannealing induces sublimation of Sb with the ratio less than 0.9 as indicated by green, yellow, and red colors. Such an Sb deficit is seen not only in the In_2_O_3_ with InSb and TiO_2_ (circle), but also in the Sb with InSb and TiO_2_ (square). Hence, the difference in phase appearance (Sb and In_2_O_3_) (Figure 4) seems to be independent of the compositional deviation from stoichiometry in a binary In-Sb system. According to the phase diagram of the In-Sb-O ternary system [19], the binary In-Sb system is in equilibrium with In_2_O_3_. A tie-line between the two phases (In_2_O_3_ and In-Sb system) indicates that the oxygen concentration dominates the phase appearance in the binary system. Specifically, relatively high oxygen content provides Sb with an InSb phase, even with a nominal Sb deficit from stoichiometric InSb. This suggestion is consistent with the present result. Sb with an InSb phase appears at relatively high oxygen concentrations exceeding 61 at.%, and less oxygen is needed to provide In_2_O_3_ with an InSb phase. It is therefore found that the difference in phase appearance (Sb and In_2_O_3_) (Figure 4) is due to the different inclusions of oxygen. In these results, the composite containing Sb does not achieve the present objective, since the residual Sb reduces the transparency. To avoid the inclusion of Sb, the sputtering target needs a different setup, such as excess In or less oxygen in the composite target, made of ceramic TiO_2_ with InSb chips. A composite with InSb and single-phase TiO_2_ cannot be obtained in the current study. However, the carrier mobility of the phase mixture of TiO_2_ and In_2_O_3_ exceeds that of the pure TiO_2_[20]. Thus, the inclusion of In_2_O_3_ is considered to be useful for the current interest.

**Figure 6:**
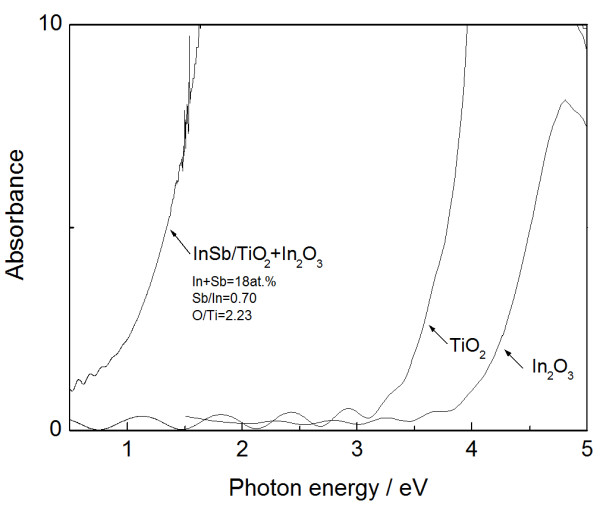
**Typical optical absorption spectra of InSb-added TiO**_**2 **_**composite film.** With a phase mixture of InSb, TiO_2_, and In_2_O_3_, containing 18 at.% (In + Sb).

**Figure 7:**
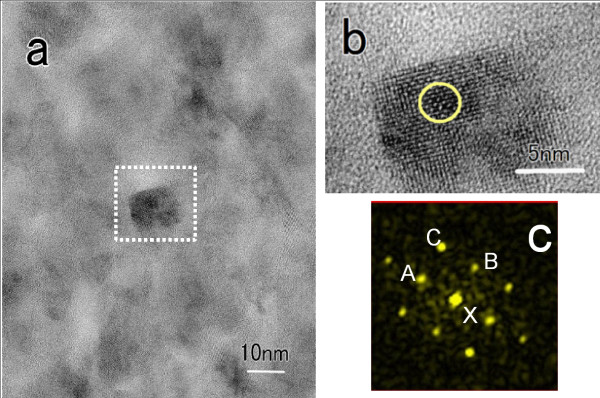
**Direct observation of InSb-added TiO**_**2 **_**nanocomposite film.** With a phase mixture of InSb, TiO_2_, and In_2_O_3_, containing 18 at.% (In + Sb). (**a**) HRTEM image. (**b**) Enlarged image for easier viewing. (**c**) FFT diffraction pattern of the selected area, indicated by the circle in (**b**).

Figure 6 depicts a typical optical absorption spectrum for composite film with InSb, TiO_2_, and In_2_O_3_. For comparison, the absorption spectra of TiO_2_ and In_2_O_3_ are also presented in the figure. The absorption edge in both TiO_2_ and In_2_O_3_ appears in the UV range, while the composite film containing 18 at.% (In + Sb) exhibits an obvious shift to the vis-NIR range, thus absorb a desirable energy region for high conversion efficiency [21]. The composite film contains Sb deficit in InSb with a ratio Sb/In of 0.7. Hence, the actual concentration of InSb compound is estimated to be 15 at.%, assuming an Sb reacts fully to form InSb compound. The film also contains excess oxygen in TiO_2_ with a ratio O/Ti of 2.23. The excess oxygen and the decomposed In may react to form In_2_O_3_. The analyzed oxygen content is enough just to form stoichiometric TiO_2_ with an estimated concentration of 76 at.% and In_2_O_3_ with 8 at.%. An HRTEM image of the composite film is presented in Figure 7a. The slightly dark sphere-like nanocrystals are clearly dispersed, with a size of approximately 15 nm. The selected area (dotted line) is enlarged in Figure 7b for easier viewing. Fast Fourier transform (FFT) analysis of the region (circle in Figure 7b) reveals the details of the local structure in the nanocrystal. Figure 7c presents the corresponding FFT diffraction pattern, which can be indexed to cubic InSb. The spots labeled A, B, and C correspond to crystal faces of (110), (1-10), and (200) in the cubic InSb, with plane widths of 0.452, 0.466, and 0.330 nm, respectively. The angles labeled A-X-B, A-X-C, and B-X-C are 89°, 46°, and 43°. The standard data (JCPDS 6–208) indicates a plane width of 0.458 nm at both (110) and (1-10), and 0.324 nm at (200), with an angle of 90° for A-X-B and 45° for both A-X-C and B-X-C. The analysis results are close to the standard data. The observed grain is thus found to be cubic InSb nanocrystal. Therefore, InSb-added TiO_2_ nanocomposite film produces a composite with InSb nanocrystals dispersed in a multiphase matrix composing TiO_2_ and In_2_O_3_. The mean grain size of the InSb nanocrystals is estimated to be 18 nm using Scherrer's formula [22] in XRD peak fitting. This size is nearly the same as that of the observed InSb nanocrystals. This is small enough to exhibit the quantum size effects because of the exciton Bohr radius of 65.5 nm in InSb [14]. Furthermore, the ground state transition of electron–hole pairs in the semiconductor nanocrystal is calculated by the following formula [23,24]: *E* = *E*_*g*_ + (*ħπ*)^2^/2*μR*^2^ − 1.8*e*^2^/4*π* ∈ ∈ _0_*R*, where *E*_*g*_ is the bulk band gap, *ħ* is the reduced Planck constant, *μ* is the reduced mass of an electron–hole pair, *R* is the effective Bohr radius, *e* is the electron charge, and ∈ is the background dielectric constant of InSb. Hence, the ground state transition of the InSb nanocrystals is calculated to be 0.78 eV, which corresponds well to the onset absorption containing 18 at.% (In and Sb) (Figure 6). Therefore, the optical absorption shift is obviously due to quantum size effects of the InSb nanocrystals embedded in the multiphase matrix, TiO_2_ and In_2_O_3_.

InSb-added Al-oxide thin film, which is a similar composite containing InSb nanocrystals, produces a mean grain size of 8 nm during postannealing at 723 K, with similar concentrations of 9.5 at.% In and 13.5 at.% Sb [25]. The present result provides InSb nanocrystals of nearly twice this size. In addition, no inclusion of In_2_O_3_ is seen in the InSb-added Al-oxide thin films, while this does appear in the present study (Figures 2 and 3). These different results are probably due to the difference in the free energy of reaction between the two oxides, TiO_2_ and Al_2_O_3_[16]. Specifically, Al_2_O_3_ with its smaller free energy of reaction is thermodynamically more stable than TiO_2_. InSb-added Al-oxide thin films also exhibit a narrower size distribution in the InSb nanocrystals compared with that of the SiO_2_ matrix [26], whose free energy of reaction is close to that of the TiO_2_. The thermodynamic stability of the matrix may affect the aggregation of the InSb nanocrystals during postannealing, although the size distribution of the InSb nanocrystals dispersed in the multiphase matrix, TiO_2_ and In_2_O_3_, is not estimated here, due to a difficulty of finding InSb nanocrystals in the HRTEM image containing three kinds of crystals, InSb, TiO_2_, and In_2_O_3_.

The present results indicate that InSb-added TiO_2_ nanocomposite films provide a composite with InSb nanocrystals embedded in a multioxide matrix composing TiO_2_ and In_2_O_3_ and exhibiting vis-NIR absorption due to quantum size effects of the InSb nanocrystals. One-step synthesis of a composite thin film therefore has potential for low-cost production of next-generation solar cells.

## Conclusions

InSb-added TiO_2_ nanocomposite films have been proposed as candidate materials for quantum dot solar cells. It should be pointed out that composite thin films with InSb nanocrystals dispersed in a multiphase composing TiO_2_ and In_2_O_3_ appear in a restricted composition range from 12 to 18 at.% (In + Sb), because of compositional variation. The optical absorption edge shifts toward the vis-NIR range, favorably absorbing a desirable energy region for high conversion efficiency. A HRTEM image indicates that the composite thin film contains spherical InSb nanocrystals with a size of approximately 15 nm. This size is sufficiently small to exhibit quantum size effects. InSb-added TiO_2_ nanocomposite films also produce In_2_O_3_, due to decomposition of the added InSb during postannealing. The electrical properties are not studied at all in the present study. However, the photocurrent of the composite may be enhanced by including In_2_O_3_, since the carrier mobility of the phase mixture of TiO_2_ and In_2_O_3_ is higher than that of the pure TiO_2_. Therefore, a multioxide matrix of TiO_2_ and In_2_O_3_ with InSb nanocrystals should be useful for next-generation solar cells.

## Competing interests

The author declares that there are no competing interests.

## Author information

SA is a group leader of the Research Institute for Electromagnetic Materials.
